# Omission of Axillary Surgery in Patients with Invasive Lobular Carcinoma of the Breast: Rates of Nodal Involvement in Clinically Node Negative Patients and Impact on Recurrence in a Single Institution Analysis

**DOI:** 10.1245/s10434-026-20144-y

**Published:** 2026-07-06

**Authors:** Elisabeth Abeles, Amy M. Shui, Astrid Quirarte, Joshua Barkin, Sarah Choi, Yanying Chen, Laura Huppert, Natalie Kim, Jackelyn Moya, Anna Vertido, Rita A. Mukhtar

**Affiliations:** 1 Division of Surgical Oncology, UCSF, San Francisco, USA; 2 Department of Epidemiology and Biostatistics, UCSF, San Francisco, USA; 3 Division of Hematology/Oncology, Department of Medicine, UCSF, San Francisco, USA; 4 Georgetown University School of Medicine, Washington, USA

## Abstract

**Background:**

Recent randomized trials such as INSEMA and SOUND identified patients with breast cancer for whom axillary surgery omission appears to have no detrimental oncologic impact. However, some have questioned the safety of omitting axillary surgery in patients with invasive lobular carcinoma (ILC), the most common special histology type.

**Methods:**

We analyzed a prospectively maintained institutional ILC database containing 1029 tumor records. We identified those meeting INSEMA and SOUND criteria for axillary surgery omission (clinically node negative with axillary ultrasound, cT1-2 for INSEMA-eligible, cT1 for SOUND-eligible, no neoadjuvant therapy). We used chi-square and Wilcoxon rank-sum tests to evaluate factors associated with pathologic nodal positivity. Local and distant recurrence free survival for those omitting axillary surgery were explored using the Kaplan-Meier method.

**Results:**

Overall, 205 patients with ILC met preoperative criteria for the INSEMA or SOUND trials, of whom 189 (92%) underwent axillary surgery. Of 189 INSEMA-eligible patients, 19.8% were node positive and 30.2% upstaged in T category. Of 99 SOUND-eligible patients, 18.4% were node positive and 34.6% upstaged in T category. Younger age was significantly associated with nodal positivity (INSEMA *p* = 0.003, SOUND *p* = 0.002). For the subset of patients who omitted axillary surgery, distant recurrence free survival was significantly worse compared to those who underwent axillary surgery in both INSEMA-eligible (*p* = 0.001) and SOUND-eligible cohorts (*p* = 0.0495).

**Conclusions:**

High rates of occult nodal positivity in ILC patients suggest the need for ILC-specific selection criteria for axillary surgery omission. Further studies are needed to evaluate potential impact on oncologic outcomes in this patient population.

Recent trials have demonstrated the safety of omitting all axillary surgery in highly selected patients with breast cancer. The Intergroup Sentinel Mamma (INSEMA) trial found that omitting sentinel lymph node biopsy (SLNB) in patients with clinically node negative, T1-2 disease did not significantly worsen invasive disease free survival, and the Sentinel Node vs Observation after Axillary Ultra-souND (SOUND) trial found that omission of SLNB in clinically node negative, T1 patients did not significantly worsen 5-year distant recurrence free survival.^1,2^ While reducing morbidity related to unnecessary axillary surgery is important, understaging the axilla might carry greater risk in certain groups of patients, such as those with invasive lobular carcinoma (ILC).

Invasive lobular carcinoma accounts for 10–15% of breast cancers and is the most common special histologic type of breast cancer. Multiple studies have demonstrated the difficulty with clinical assessment of disease burden in patients with ILC. Invasive lobular carcinoma lacks the adhesion protein E-cadherin and consequently grows in a linear pattern, which can be difficult to identify on physical exam and imaging.^3,4^ Because ILC can spread diffusely throughout the breast and even to lymph nodes without forming an identifiable mass, diagnosis is often delayed and upstaging on pathology is common.^5–7^ With these well-known challenges of accurately determining clinical tumor and nodal stage, the impact of omitting surgical staging of the axilla in those with ILC is not well understood. Indeed, a recent analysis of 491 patients with cT1N0 ILC who met SOUND trial criteria found that 15.9% had occult axillary nodal macrometastases compared with 8.6% of those reported in SOUND, which largely enrolled patients with carcinoma of no special type (NST).^8^ As of yet, there are no reported data evaluating the impact of axillary surgery omission on patients with ILC who meet INSEMA and/or SOUND trial criteria. In this analysis, we report rates of nodal involvement, factors associated with node positivity, tumor upstaging rates, and explored the impact of axillary surgery omission on local and distant recurrence in a single institution cohort of clinically node negative patients with ILC. These data may help to guide selection of ILC patients for whom SLNB omission can be considered.

## Methods

### Study Design

We analyzed a prospectively maintained institutional database of patients with ILC containing 1029 tumor records from 1001 patients who underwent surgery between 1987 and 2025. Twenty-eight patients (2.8%) had bilateral disease. All tumor records were analyzed as independent observations.

### Inclusion and Exclusion Criteria

After excluding 18 patients with stage 4 disease at diagnosis, we categorized the remaining 1,011 ILC cases into three groups: 1) those that met INSEMA trial criteria; 2) those that met SOUND trial criteria; and 3) those that met neither. The INSEMA-eligible group were all clinically node negative based on preoperative axillary ultrasound, had clinical tumor size ≤ 5 cm and did not receive neoadjuvant therapy. The SOUND-eligible group met the same criteria, with the more restrictive clinical tumor size requirement being ≤ 2 cm. Of note, all patients considered SOUND-eligible were also considered INSEMA-eligible.

Axillary ultrasound was required to define clinical nodal status, with our standard institutional approach being to perform needle biopsy for axillary nodes with cortical thickness of ≥3 mm. Those with positive needle biopsy were considered clinically node positive (cN+). At surgery, those with one or more lymph nodes containing macrometastasis (tumor deposit > 2 mm) were considered pathologic node positive (pN+), whereas those with negative nodes, isolated tumor cells, or micrometastatic disease were considered pathologic node negative.

Clinical and pathologic tumor staging was determined by using the *American Joint Commission on Cancer Staging Manual,* 7th edition criteria.^9^ For clinical tumor staging, largest tumor size in centimeters (cm) by physical examination or imaging and/or documented T category was recorded from the medical record. Of note, our institutional practice is to obtain preoperative magnetic resonance imaging (MRI) of the breast for patients with ILC, barring a clinical contraindication. For pathologic tumor staging, continuous tumor size (cm) was recorded from the surgical pathology report.

### Clinicopathologic Variables

We analyzed variables including age at diagnosis, menopausal status, tumor grade (1–3), presence of pleomorphic histologic subtype, tumor proliferation rate defined by percent positivity for Ki67, presence of lymphovascular invasion, and presence of extranodal extension in those with node positive disease. Tumor receptor subtype was defined by immunohistochemistry (IHC) for estrogen receptor (ER), progesterone receptor (PR), and human epidermal receptor growth factor receptor 2 (HER2); HER2 status is also routinely confirmed by fluorescence in situ hybridization (FISH) at our institution. ER and PR positivity were defined as >1% staining on IHC, and ER- or PR-positive tumors were considered hormone receptor (HR)-positive. Type of locoregional therapy was collected including lumpectomy alone, lumpectomy with radiation (breast conservation therapy [BCT]), mastectomy alone, or mastectomy with radiation. Type of axillary surgery was recorded; of note, a subset of patients underwent breast surgery without axillary nodal surgery.

### Statistical Approach

In the INSEMA-eligible and SOUND-eligible cohorts, we determined the proportion of patients who were found to be pathologic node positive and evaluated factors associated with pN+ status using chi-square and Wilcoxon rank-sum tests. Additionally, we determined the proportion of patients who had upstaging in tumor category (higher pT category than cT category).

We evaluated recurrence outcomes using the Kaplan-Meier method. We evaluated local recurrence free survival (LRFS), defined as the absence of locoregional recurrence, and distant recurrence free survival (DRFS), defined as the absence of distant recurrence; patients were censored at the time of recurrence or at loss to follow up. Because a subset of patients in this study did not have axillary surgery, LRFS and DRFS were compared in the following groups: 1) INSEMA-eligible patients with and without omission of axillary surgery; 2) SOUND-eligible patients with and without axillary surgery. We further evaluated LRFS and DRFS only among the subset of INSEMA-eligible patients and SOUND-eligible patients undergoing BCT (lumpectomy with radiation). We tested differences between groups with log-rank tests; survival analysis were right censored at 15 years from the date of diagnosis.

All variables used in bivariate tests had <5% missing data, hypothesis tests were two-sided, and the significance threshold was set to 0.05. Statistical analysis was performed by using SAS version 9.4.

## Results

Of 1,011 cases of ILC, 205 (20.3%) were INSEMA-eligible, of whom 109 (10.8% of total study cohort) also met the more restrictive SOUND-eligibility criteria (Table 1). The remaining 806 cases (79.7%) were not eligible for either study.

**Table 1:** Patient characteristics of SOUND-eligible and INSEMA-eligible cohorts

Characteristic	INSEMA-eligible *n* = 205	SOUND-eligible *n* = 109
**Patient characteristics**		
Age at diagnosis, years (median (range))	61 (21–89)	61 (21–89)
Postmenopausal^a^		
Yes	141 (70.5%)	75 (70.8%)
No	59 (29.5%)	31 (29.2%)
**Type of surgery**^**b**^		
Lumpectomy + radiation	97 (49.2%)	53 (50.5%)
Lumpectomy alone	31 (15.7%)	20 (19%)
Mastectomy + radiation	11 (5.6%)	1 (1%)
Mastectomy alone	58 (29.4%)	31 (29.5%)
**Tumor characteristics**		
Hormone receptor subytpe^c^		
HR+HER2−	187 (93%)	99 (91.7%)
HER2+	10 (5%)	8 (7.4%)
ER−PR−HER2−	4 (2%)	1 (0.9%)
Lymphovascular invasion		
Yes	11 (5.4%)	5 (4.6%)
No	194 (94.6%)	104 (95.4%)
Ki-67 mean, median (range)	11, 10 (1–50)	10.5, 10 (1–50)
Grade^d^		
1	43 (21.1%)	25 (23.1%)
2	144 (70.6%)	78 (72.2%)
3	17 (8.3%)	5 (4.6%)
cT, cm^e^ (mean, median (range))	2.3, 2.0 (0.2–5.0)	1.4, 1.5 (0.2–2.0)
Clinical tumor stage		
cT1	109 (53.2%)	109 (100%)
cT2	96 (46.8%)	0 (0%)
cT3	0 (0%)	0 (0%)
pT, cm^f^ (mean, median (range))	2.8, 2.2 (0.2–12.9)	1.9, 1.5 (0.2–8.0)
Pathologic tumor stage		
pT1	96 (47.5%)	70 (65.4%)
pT2	77 (38.1%)	32 (29.9%)
pT3	29 (14.4%)	5 (4.7%)
Axillary surgery		
Yes	189 (92.2%)	99 (90.8%)
No	16 (7.8%)	10 (9.2%)
Positive LN after axillary surgery^g^		
Yes	37 (19.8%)	18 (18.4%)
No	150 (80.2%)	80 (81.6%)
# positive nodes (mean, median (range))	2, 1 (1–15)	2, 1 (1–8)
Nodal category (among node positive cases)		
pN1	33 (89.2%)	16 (88.9%)
pN2	3 (8.1%)	2 (11.1%)
pN3	1 (2.7%)	0 (0%)
Extranodal/extracapsular extension		
Yes	6 (2.9%)	2 (1.8%)
No	199 (97.1%)	107 (98.2%)

Total overall INSEMA-eligible *n* = 205, SOUND-eligible *n* = 109 unless otherwise specified

*ER* estrogen receptor; *PR* progesterone receptor; *HER2* human epithelial growth factor receptor-2; *cT* clinical tumor size; *pT* pathologic tumor size; *LN* lymph node; *pN* pathologic node status

^a^Data available for 200 INSEMA cases; for 106 SOUND

^b^Data available for 197 INSEMA cases; for 105 SOUND

^c^Data available for 201 INSEMA cases, 108 INSEMA cases

^d^Data available for 204 INSEMA cases, 108 SOUND cases

^e^Data available for 203 INSEMA cases, 109 SOUND cases

^f^Data available for 202 INSEMA cases, 107 SOUND cases

^g^Data available in 187 INSEMA cases, 98 SOUND cases

### INSEMA-Eligible Cohort

Of the 205 patients who met INSEMA criteria, age at diagnosis ranged from 21–89 years (median 61); 141 (70.5%) patients were postmenopausal. Most patients (*n* = 144; 70.6%) had grade 2 disease. Median Ki-67 was 10% (range 1–50%), and 11 (5.4%) had lymphovascular invasion. Most tumors 187 (93%) were HR+HER2−; ten (5%) were HER2+, and four (2%) were triple negative. Locoregional therapy was lumpectomy with radiation in 97 (49.2%), mastectomy in 58 (29.4%), lumpectomy alone in 31 (15.7%), and mastectomy with radiation in 11 (5.6%). Sixteen (7.8%) patients omitted axillary surgery.

### SOUND-Eligible Cohort

Of the 109 patients who met SOUND criteria, age at diagnosis ranged from 21–89 (median 61); 75 (70.8%) were postmenopausal. Most patients, 78 (72.2%) had grade 2 disease. Median Ki-67 was 10% (range (1–50%), and five (4.6%) had lymphovascular invasion. Most tumors (*n* = 99; 91.7%) were ER+HER2−; eight (7.4%) were HER2+, and 0.9% were triple negative. Overall, 53 (50.5%) underwent lumpectomy with radiation, 31 (29.5%) had mastectomy alone, 20 (19.0%) underwent lumpectomy alone, and one (1%) had mastectomy with radiation. Ten (9.2%) patients omitted axillary surgery.

### Rates of Nodal Positivity in INSEMA-Eligible and SOUND-eligible Cohorts

**Fig. 1:**
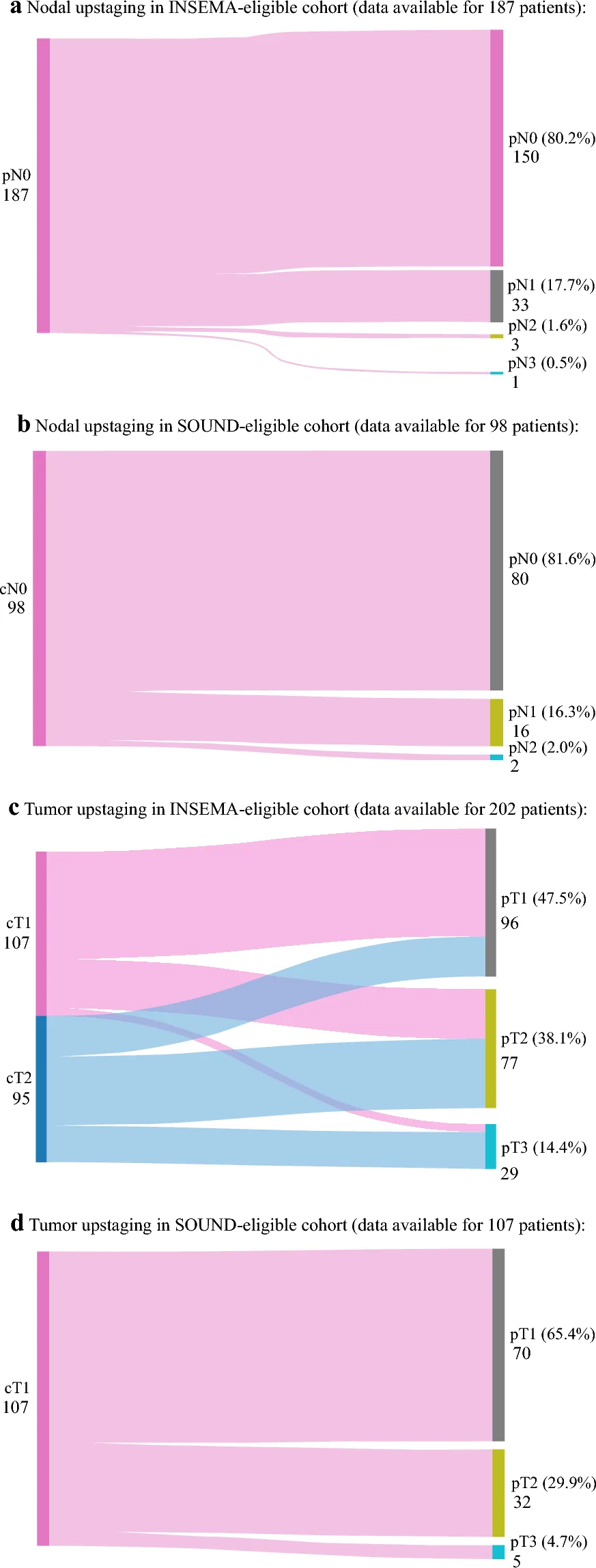
Sankey diagrams displaying nodal and tumor upstaging for patients in INSEMA-eligible and SOUND-eligible cohorts who had both clinical and pathologic tumor size/node data available. **a** Nodal upstaging in INSEMA-eligible cohort (data available for 187 patients). **b** Nodal upstaging in SOUND-eligible cohort (data available for 98 patients). **c** Tumor upstaging in INSEMA-eligible cohort (data available for 202 patients). **d** Tumor upstaging in SOUND-eligible cohort (data available for 107 patients)

In the 189 patients who met INSEMA criteria and had axillary surgery, the nodal positivity rate on pathology was 19.8% (Fig. 1a). The mean number of positive nodes was 2.2 (range 1–15 and median 1 node). Of these pN+ patients, 33 (89.2%) were pN1, three (8.1%) were pN2, and one (2.7%) was pN3.

In the 99 patients who met SOUND criteria and had axillary surgery, the nodal positivity rate on pathology was 18.4% (Fig. 1b). The mean number of positive nodes was two (range 1–8, and median 1 node). Of these pN+ patients, 16 (88.9%) were pN1, two (11.1%) were pN2, and no patients upstaged to pN3 disease.

### Factors Associated with Nodal Positivity

In both INSEMA-eligible and SOUND-eligible cohorts, the only factor significantly associated with nodal positivity at surgery was younger age (Table 2). For the INSEMA-eligible cohort*,* median age was significantly lower in pN+ patients compared with pN− patients (53 vs. 61 years, *p* = 0.03). Among patients <60 years, the rate of pN+ status was 26%; among those <50 years, the rate of pN+ status was 28%. However, pathologic tumor size, receptor subtype, tumor grade, presence of LCIS, pleomorphic histology, multifocality, LVI, and menopausal status were not significantly associated with nodal positivity.

**Table 2:** Factors associated with nodal positivity

	Node-positive; median age (range)	Node-negative; median age (range)	*p*
INSEMA-eligible, *n* = 205	53 (41–86)	61 (21–89)	0.03
SOUND-eligible, *n* = 109	51 (41–76)	62.5 (21–89)	0.02

For the SOUND-eligible cohort median age in pN+ versus pN− patients was 51 years versus 62.5 years (*p* = 0.02). Among patients <60 years, the rate of pN+ status was 25%; among those <50 years, the rate of pN+ status was 33%. However, pathologic tumor size, receptor subtype, tumor grade, presence of LCIS, pleomorphic histology, multifocality, LVI, and menopausal status were not significantly associated with nodal positivity.

### Rates of Tumor Upstaging in INSEMA-Eligible and SOUND-Eligible Cohorts

In patients who met INSEMA trial criteria, mean clinical tumor size was 2.3 cm (range 0.2–5.0 cm). Overall, 109 (53.2%) had cT1 disease, and 96 (46.8%) had cT2 disease. In contrast, mean pathologic tumor size was 2.8 cm (range 0.2–12.9 cm); most patients (*n* = 96; 47.5%) were pT1, 77 (38.1%) were pT2, and 29 (14.4%) were pT3. Of these patients, 32 (15.8%) patients upstaged from cT1 to pT2, five patients from cT1 to pT3 (2.5%), and 24 (11.9%) patients from cT2 to pT3. In total, 61 (30.2%) patients upstaged in T category (Fig. 1c).

In patients who met the more restrictive SOUND criteria, mean clinical tumor size was 1.4 cm (range 0.2–2.0 cm); all 109 (100% of cases) had cT1 tumors. In this group, mean pathologic tumor size was 1.9 cm (range 0.2–8.0 cm), with pT1 in 70 (65.4%), pT2 in 32 (29.9%), and pT3 in 5 (4.7%). In total, 37 patients (34.6%) upstaged in T category (Fig. 1d).

### Association Between Omission of Axillary Surgery and Recurrence Outcomes

Because a subset of patients in these cohorts did not undergo axillary surgery, we explored the relationship between axillary surgery omission and time to recurrence. We evaluated this in the following groups: all INSEMA-eligible patients, INSEMA-eligible patients who underwent BCT, all SOUND-eligible patients, and SOUND-eligible patients who underwent BCT.

**Fig. 2:**
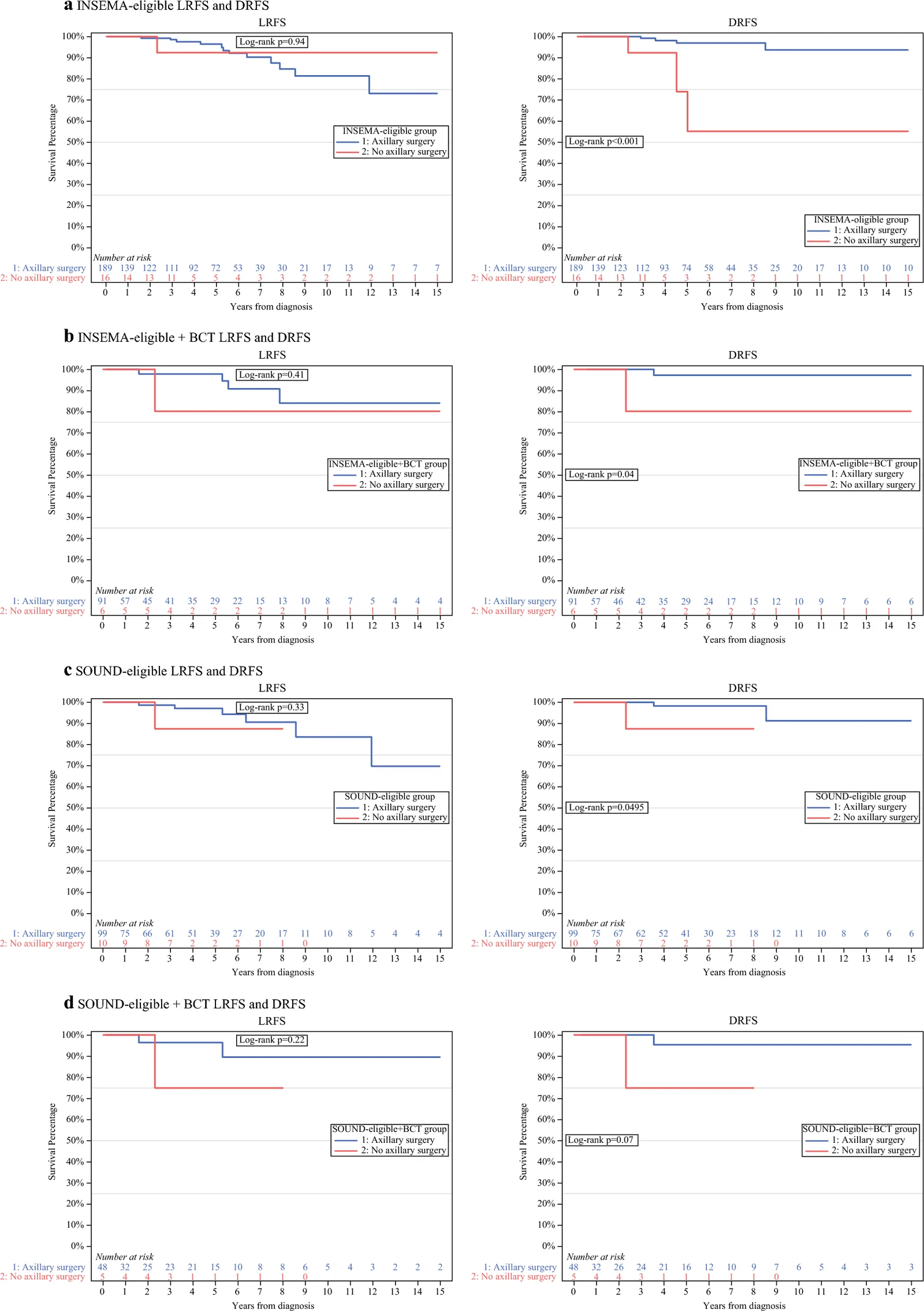
Local recurrence-free survival (LRFS) and distant recurrence-free survival (DRFS) in INSEMA-eligible patients, INSEMA-eligible patients who underwent breast-conserving therapy (BCT), SOUND-eligible patients, and SOUND-eligible patients who underwent BCT. **a** INSEMA-eligible LRFS and DRFS. **b** INSEMA-eligible + BCT LRFS and DRFS. **c** SOUND-eligible LRFS and DRFS. **d** SOUND-eligible + BCT LRFS and DRFS

Among all INSEMA-eligible patients, there was so significant difference in LRFS between the 189 cases who had axillary surgery and the 16 cases who did not have axillary surgery (*p* = 0.94). At 15 years, the estimated cumulative incidence of local recurrence was 26.9% in the axillary surgery group (95% confidence interval [CI] 8.6–45.2%) and 7.7% in the no axillary surgery group (95% CI −6.8–22.2%), with a total of 13 local recurrence events. However, there was a significant association between omission of axillary surgery and reduced DRFS (*p* < 0.001). Among those undergoing axillary surgery, the estimated cumulative incidence of distant recurrence by 15 years was 6.5% (95% CI −0.9–14%) compared with 44.6% (95% CI 4–85.2%) in the no axillary surgery group, with a total of seven events (Fig. 2a).

Among INSEMA-eligible patients who underwent BCT, there was so significant difference in LRFS between the 91 cases who had axillary surgery and the six cases who did not have axillary surgery (*p* = 0.41). At 15 years, the estimated cumulative incidence of local recurrence was 15.8% in the axillary surgery group (95% CI 0.2–31.4%) and 20% in the no axillary surgery group (95% CI −15–55%), with a total of five local recurrence events. However, there was a significant association between omission of axillary surgery and reduced DRFS (*p* = 0.04). Among those undergoing axillary surgery, the estimated cumulative incidence of distant recurrence by 15 years was 2.6% (95% CI −2.5–7.7%) compared with 20% (95% CI −15–55.1%) in the no axillary surgery group, with a total of two events (Fig. 2b).

Among all SOUND-eligible patients, there was so significant difference in LRFS between the 99 cases who had axillary surgery and the ten cases who did not have axillary surgery (*p* = 0.33). At 15 years, the estimated cumulative incidence of local recurrence was 30.4% in the axillary surgery group (95% CI 2.2–58.5%) and 12.5% in the no axillary surgery group (95% CI −10.4–35.4%), with a total of only seven local recurrence events. In these patients, however, axillary surgery was associated with improved DRFS (*p* = 0.0495). Among those undergoing axillary surgery, the estimated cumulative incidence of distant recurrence by 15 years was 8.8% (95% CI −4.9–22.4%) compared with 12.5% (95% CI −10.4–35.4%) in the no axillary surgery group, with a total of three events (Fig. 2c).

Among SOUND-eligible patients who underwent BCT, there was so significant difference in LRFS between the 48 cases who had axillary surgery and the five cases who did not have axillary surgery (*p* = 0.22). At 15 years, the estimated cumulative incidence of local recurrence was 10.6% in the axillary surgery group (95% CI −4–25.2%) and 25% in the no axillary surgery group (95% CI −17.4–67.4%), with a total of only three local recurrence events. Similarly, there was no difference in DRFS (*p* = 0.07), with estimated cumulative incidence of distant recurrence of 4.6% (95% CI −4.2–13.3%) in the axillary surgery group, and 25% (95% CI −17.4–67.4%) in the no axillary surgery group, with a total of two events (Fig. 2d).

Across all four of these patient groups, the proportion who received adjuvant endocrine therapy was similar regardless of whether axillary surgery was omitted (INSEMA eligible: 82.3% vs. 75%, *p* = 0.47; INSEMA eligible+BCT: 92.2% vs. 83.3%, *p* = 0.45; SOUND eligible: 78.4% vs. 80%, *p* = 0.90; SOUND eligible+BCT: 89.4% vs. 80%, *p* = 0.53). The proportion who received radiation was also similar regardless of whether axillary surgery was omitted in both the INSEMA-eligible and SOUND-eligible groups (INSEMA eligible: 54.8% vs. 43.8%, *p* = 0.39; SOUND eligible: 50.5% vs. 50%, *p* = 0.98), whereas all patients in the BCT subgroups received radiation regardless of axillary surgery omission or inclusion (Tables 3a–d).

**Table 3:** Adjuvant endocrine therapy, radiation, and age by axillary surgery status (chi-square and Wilcoxon rank-sum tests)

	Axillary surgery (*n* = 189)	No axillary surgery (*n* = 16)	*p*
a: INSEMA-eligible patients, *n* = 205			
Age, median (range)	59.0 (21–89)	71.5 (48–88)	<0.001
Received adjuvant endocrine therapy	153/186 (82.3%)	12/16 (75%)	0.47
Received adjuvant radiotherapy	102/186 (54.8%)	7/16 (43.8%)	0.39

	Axillary surgery (*n* = 91)	No axillary surgery (*n* = 6)	*p*
b: INSEMA-eligible + BCT patients, *n* = 97			
Age, median (range)	58.0 (21–82)	76.5 (63–83)	0.002
Endocrine therapy, Yes	83/90 (92.2%)	5/6 (83.3%)	0.45
Radiation, Yes	91/91 (100%)	6/6 (100%)	N/A

	Axillary surgery (*n* = 99)	No axillary surgery (*n* = 10)	*p*
c: SOUND-eligible patients, *n* = 109			
Age, median (range)	59.0 (21–89)	71.5 (58–88)	0.005
Endocrine therapy, Yes	76/97 (78.4%)	8/10 (80%)	0.90
Radiation, Yes	49/97 (50.5%)	5/10 (50%)	0.98

	Axillary surgery (*n* = 48)	No axillary surgery (*n* = 5)	*p*
d: SOUND-eligible + BCT patients, *n* = 53			
Age, median (range)	58.5 (21–80)	80.0 (63–83)	0.005
Endocrine therapy, yes	42/47 (89.4%)	4/5 (80%)	0.53
Radiation, yes	48/48 (100%)	5/5 (100%)	N/A

## Discussion

In this single institution analysis of 1,029 cases of ILC, we found that 205 patients met preoperative INSEMA criteria and 109 met SOUND criteria, making them potentially eligible for axillary surgery omission. Unexpected nodal macrometastases were present in 19.8% of INSEMA-eligible patients and 18.4% of SOUND-eligible patients in this study, in contrast to the reported nodal positivity rates of 11.4% in the INSEMA trial and 8.6% in the SOUND trial.^1,2^ Nodal positivity was significantly more common in younger women, and approximately one-third of the patients in this cohort had upstaging of tumor category at surgery. Importantly, we also found a significant association between omission of axillary surgery and shorter DRFS in our patient population; while exploratory in nature due to the small number of patients who omitted axillary surgery and the retrospective nature of this analysis, this finding raises the possibility of detrimental oncologic outcomes in patients with ILC who omit axillary surgery

The nodal positivity rate in our analysis is consistent with a recent publication from Scardina et al., who found a nodal upstaging rate of 20.2% in a cohort of patients with ILC meeting SOUND trial criteria.^8^ Numerous studies have shown high rates of nodal upstaging in those with ILC. In a study of 349 patients with ILC, Schumaker et al. found significant discordance clinical and pathologic N stage; while 70.5% of their cohort was cN0, only 62% were pN0 despite preoperative axillary imaging.^10^ Similarly, in a study of 692 ILC patients, Cocco et al. found a 30% rate of nodal upstaging from cN0 to at least pN1, with 6.7% upstaging to pN2/N3 disease.^11^ Occult nodal disease in ILC is often attributed to its single-file growth pattern, which can result in minimal change to lymph node architecture, as opposed to the standard findings of enlarged, rounded, and cortically thickened nodes in patients with NST.^12,13^ Indeed, axillary ultrasound has been shown to have lower sensitivity for nodal involvement in patients with ILC, with sensitivity ranging from 32–52%, versus 50–89% for those with NST.^14^

We found a significant association between younger age and nodal positivity in our cohort. Surprisingly, for those <60 years of age meeting INSEMA/SOUND criteria, approximately one of four patients were node positive. For those <50 years of age, one of three patients were node positive. This finding is consistent with prior studies showing that younger women are more likely to have nodal involvement or more advanced nodal disease in general.^15–17^ Because of the concern for missing nodal involvement and potentially undertreating younger women, current guidelines suggest omitting SLN surgery only in those aged 50 years or older.^18,19^ For patients with ILC, this could result in up to 25% chance of missed nodal disease.

While we did not identify an association between larger tumor size and nodal involvement, we did note a high rate of tumor upstaging, with approximately 30% of all INSEMA and SOUND eligible patients had increase in T category. This finding has also been previously reported in the literature; many studies showed that conventional imaging tends to underestimate size of ILC, while breast magnetic resonance imaging (MRI) has the greatest sensitivity for this tumor type.^20,21^ While breast MRI is typically utilized for the preoperative evaluation of patients with lobular histology at our institution, we did not have MRI data available for this analysis.

Importantly, in our cohort, the omission of axillary surgery for INSEMA-eligible and SOUND-eligible patients with ILC was significantly associated with decreased DRFS. This differs from INSEMA trial and SOUND trial, which found no detrimental effect in invasive disease-free survival and 5-year DRFS, respectively.^1,2^ This significant association between axillary surgery omission and worse DRFS persisted in the subset of INSEMA-eligible patients who had lumpectomy with radiation, suggesting that the addition of radiation did not compensate for omission of axillary surgery for this small cohort of ILC patients. However, this finding should be interpreted with caution, because only 8% of the study population omitted axillary surgery, limiting sample size and potentially introducing bias.

The recurrence analysis was limited by the small size of the cohort in whom axillary surgery was not performed, along with the small number of outcome events that prevented multivariable modeling, and wide confidence intervals. Given these limitations, our analysis was exploratory and hypothesis-generating. Because this is a retrospective analysis, we cannot conclude that omission of axillary surgery led to worse disease-free survival outcomes. It is possible that patients who chose to omit axillary surgery also omitted other aspects of treatment, such as systemic therapy. While we did not find a statistically significant difference in the use of adjuvant endocrine therapy in those who underwent versus omitted axillary surgery, our analysis is limited by lack of data on type and duration of therapy. It is also possible that the absence of nodal staging led to undertreatment, both in regard to extent of radiotherapy and use of CDK4/6 inhibitors.^22^ For example, the phase III MONARCHE and NATALEE trials have demonstrated that the addition of the CDK4/6 inhibitors abemaciclib and ribociclib, respectively, reduced the chance of recurrence in patients with high risk node negative and node positive HR+/HER2− breast cancer; accurate nodal staging is critical to determine eligibility for these adjuvant therapies, especially for those with ILC.^23–25^ Therefore, for patients with a higher baseline risk of nodal involvement and higher chance of occult nodal disease, such as those with lobular histology, the benefit of omitting axillary staging must be weighed against the potential cost of missing disease and potentially forgoing adjuvant radiation or systemic therapies.

Conversely, most patients in this study cohort did not have nodal involvement and therefore should not benefit from axillary surgery. With improved patient selection, we might be able to identify these patients more accurately. Patient age could help to risk stratify patients with ILC, as prior investigators have demonstrated the safety of applying Choosing Wisely guidelines to patients with ILC over the age of 70 years.^26,27^ Further investigation into novel imaging tools, such as fluoroestradiol based positron emission tomography might provide novel ways to predict which patients with ILC can safely omit axillary surgery.^28,29^

Additional limitations of this study include its single-center design, which might limit generalizability, and the lack of specific radiotherapy data including treatment fields.

## Conclusions

We found high rates of occult nodal involvement and tumor upstaging in patients with ILC who would have met criteria for the INSEMA or SOUND trials. We observed significantly worse distant recurrence-free survival in the small group of patients who omitted axillary surgery in this cohort, a novel finding that raises the possibility that axillary surgery omission might be more consequential for those with ILC than carcinoma of no special type. These findings highlight the need for ILC specific analyses in clinical trials, as well as the importance of long-term follow-up, as ILC is known to have risk of late recurrence.^30,31^ While certain patients with ILC may opt for omission of axillary surgery, understanding the potential risk of occult nodal disease for patients with this tumor type may help with shared decision making in this unique patient population. Based on these findings, it may be prudent to utilize a higher age threshold for recommending omission of axillary surgery in patients with ILC until further data are available.
